# Changes in Use of Tobacco and Alcohol During the COVID-19 Pandemic

**DOI:** 10.1192/j.eurpsy.2022.622

**Published:** 2022-09-01

**Authors:** E. Fadeeva, K. Vyshinsky, T. Klimenko

**Affiliations:** 1 National Medical Research Centre for Psychiatry and Narcology n.a. V.Serbsky Russian Federation Ministry of Health, Department Of Preventive Care In Narcology, Moscow, Russian Federation; 2 National Research Centre on Addictions – branch, Federal State Budgetary Institution «National Medical Research Center for Psychiatry and Narcology named after VP Serbsky» of the Ministry of Health of the Russian Federation, Epidemiology Department, Moscow, Russian Federation; 3 National Research Centre on Addictions – branch, Federal State Budgetary Institution «National Medical Research Center for Psychiatry and Narcology named after VP Serbsky» of the Ministry of Health of the Russian Federation, Directorate, Moscow, Russian Federation

**Keywords:** COVID-19 and substance use, cigarette smoking, alcohol use, wastewater-based methods

## Abstract

**Introduction:**

The survey assessed changes in tobacco, alcohol and other substance use during the COVID-19 pandemic.

**Objectives:**

The survey was carried out in Moscow and Nizhegorodskaya Oblast in December, 2020 - February, 2021 and included 650 medical organizations’ employees and 344 individuals with harmful alcohol or other substances use.

**Methods:**

The instrument included ASSIST, Kessler-10 and IES-R tests modified for self-reporting about different pandemic periods.

**Results:**

Among medical workers 36.8% smoked last 12 months; during the COVID-19 pandemic 13% maintained usual cigarette smoking level, 2.4% increased smoking during incidence rises. 71.2% drank alcohol last 12 months; during incidence rises 20.4% drank as usual, 15.0% drank less frequently; 2.4% increased frequency of drinking, 1.8% volumes on drinking days, 1.3% frequency of heavy episodic drinking. In harmful substance use group 61.9% smoked last 12 months; during COVID-19 incidence rises 40% kept their usual level of smoking; 13.4% increased their smoking during the first and 8.7% during the second ‘wave’ of the pandemic. 90.1% drank alcohol last 12 months; during incidence rises 49% kept drinking as usual, 20% reduced drinking and 17.3% increased drinking frequency, 21.0% volumes on drinking days, 16.4% heavy episodic drinking frequency. Wastewater-based epidemiology analysis performed in Moscow Oblast location demonstrated significant increase during COVID-19 pandemic, compared to same period 2 years earlier: inhaled nicotine use by average of 40%, ethanol consumption by average of 49%.

**Conclusions:**

Changes in cigarette smoking and alcohol use during the COVID-19 pandemic had significant variation. Increases were more likely to occur during the pandemic ‘waves’ among individual from harmful users’ group.

**Disclosure:**

No significant relationships.

